# Mechanisms of Insulin Resistance at the Crossroad of Obesity with Associated Metabolic Abnormalities and Cognitive Dysfunction

**DOI:** 10.3390/ijms22020546

**Published:** 2021-01-07

**Authors:** Thomas M. Barber, Ioannis Kyrou, Harpal S. Randeva, Martin O. Weickert

**Affiliations:** 1 Warwickshire Institute for the Study of Diabetes, Endocrinology and Metabolism, University Hospitals Coventry and Warwickshire, Clifford Bridge Road, Coventry CV2 2DX, UK; t.barber@warwick.ac.uk (T.M.B.); kyrouj@gmail.com (I.K.); harpal.randeva@uhcw.nhs.uk (H.S.R.); 2 Division of Biomedical Sciences, Warwick Medical School, University of Warwick, Coventry CV2 2DX, UK; 3 Aston Medical Research Institute, Aston Medical School, College of Health and Life Sciences, Aston University, Birmingham B4 7ET, UK; 4 Centre for Sport, Exercise and Life Sciences, Faculty of Health & Life Sciences, Coventry University, Coventry CV1 5FB, UK

**Keywords:** insulin resistance, obesity, metabolic dysfunction, cognitive dysfunction

## Abstract

Obesity mediates most of its direct medical sequelae through the development of insulin resistance (IR). The cellular effects of insulin occur through two main postreceptor pathways that are the phosphatidylinositol 3-kinase (PI3-K) and the mitogen-activated protein kinase (MAP-K) pathways. Obesity-related IR implicates the PI3-K pathway that confers the metabolic effects of insulin. Numerous and complex pathogenic pathways link obesity with the development of IR, including chronic inflammation, mitochondrial dysfunction (with the associated production of reactive oxygen species and endoplasmic reticulum stress), gut microbiota dysbiosis and adipose extracellular matrix remodelling. IR itself plays a key role in the development of metabolic dysfunction, including hypertension, dyslipidaemia and dysglycaemia. Furthermore, IR promotes weight gain related to secondary hyperinsulinaemia, with a resulting vicious cycle of worsening IR and its metabolic sequelae. Ultimately, IR underlies obesity-related conditions such as type 2 diabetes mellitus (T2D) and polycystic ovary syndrome (PCOS). IR also underlies many obesity-related malignancies, through the effects of compensatory hyperinsulinaemia on the relatively intact MAP-K insulin pathway, which controls cellular growth processes and mitoses. Furthermore, the emergent data over recent decades support an important role of obesity- and T2D-related central IR in the development of cognitive dysfunction, including effects on hippocampal synaptic plasticity. Importantly, IR is largely reversible through the optimisation of lifestyle factors that include regular engagement in physical activity with the avoidance of sedentariness, improved diet including increased fibre intake and sleep sufficiency. IR lies at the key crossroad between obesity and both metabolic and cognitive dysfunction. Given the importance of IR in the pathogenesis of many 21st century chronic diseases and its eminent reversibility, it is important that we all embrace and facilitate optimised lifestyles to improve the future health and wellbeing of the populace.

## 1. Introduction

The global obesity epidemic has developed over the last half century [1]. Obesity underlies many 21st century chronic diseases and confers a substantial socio-economic burden globally, including contributing towards a large proportion of overall healthcare costs [1]. Obesity affects people of any age, class, ethnicity or socio-economic group, with a current prevalence in the UK of one in four adults [1].

Weight gain and obesity mediates most of its direct medical sequelae through worsening insulin sensitivity and, as such, the development of insulin resistance (IR) [2]. In addition to malignancies such as endometrial carcinoma [1,3], IR underlies the cardio-metabolic dysfunction that associates with obesity. This includes type 2 diabetes mellitus (T2D) [1], polycystic ovary syndrome (PCOS) [4,5] and hypertension [6]. Furthermore, such obesity-related IR is compounded by the development of other obesity-related conditions such as obstructive sleep apnoea (OSA) [7] and male obesity-associated secondary hypogonadism (MOSH) [8]. As such, IR lies at a key junction that is itself influenced by multiple and complex pathways but also instigates multiple and complex downstream effects that ultimately manifest with metabolic and cognitive dysfunction.

**Figure 1:**
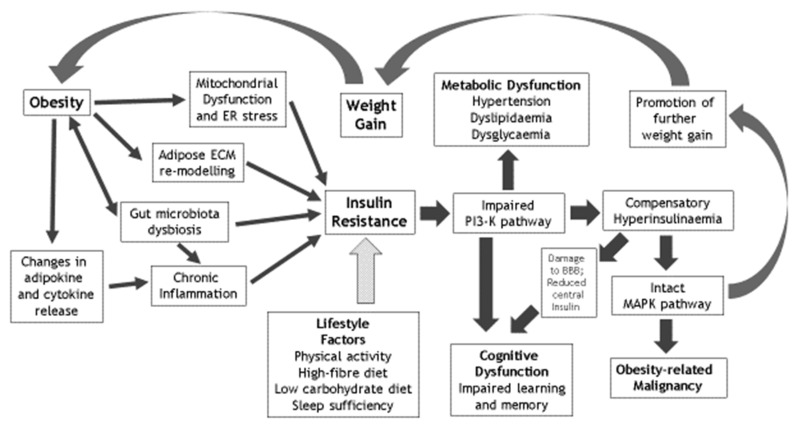
Overview of the mechanisms that underlie obesity-related insulin resistance (IR) and the metabolic and cognitive sequelae of IR. BBB: blood brain barrier; ECM: extracellular matrix; ER: endoplasmic reticulum; MAPK: mitogen-activated protein kinase; PI3K: phosphotidylinositol 3-kinase. Solid arrows: causative factors; speckled grey arrow: inhibitory factors.

In this concise review (summarised in Figure 1), we explore the role of weight gain with excess adiposity and obesity in the development of IR and the effects of IR on the subsequent development of both metabolic and cognitive dysfunction. We also consider the reversibility of IR through the effective implementation of lifestyle improvement strategies including optimisation of physical activity, diet and sleep. Although inherent challenges limit the successful longer-term maintenance of body weight following initial weight loss [9,10], successful adoption of a healthy lifestyle regarding these key lifestyle factors (regardless of the magnitude of weight loss) provides a sensible and feasible means of improving insulin sensitivity and the overall health and wellbeing of the populace.

## 2. Role of Obesity in the Development of IR

The clinical definition of IR is the inability of a known quantity of insulin (either endogenous or exogenous) to increase the uptake and utilisation of glucose in an individual by the same degree as that in a normal population [11]. Insulin binds to its plasma membrane receptor and mediates its cellular effects through a series of protein–protein interactions [11]. There are two main postreceptor cellular pathways implicated: (i) the phosphatidylinositol 3-kinase (PI3-K) pathway and (ii) the mitogen-activated protein kinase (MAP-K) pathway [12,13]. Each of these pathways confers different cellular functions of insulin. The PI3-K pathway regulates cellular intermediary metabolism, whereas the MAP-K pathway controls growth processes and mitoses [11]. Importantly, obesity-related IR appears to affect predominantly and commensurately the PI3-K pathway, with the MAP-K pathway remaining relatively unaffected [11,12,13,14]. Therefore, a more descriptive definition of IR is perhaps “metabolic insulin resistance” [14]. The relative sparing of the MAP-K insulin pathway in obesity-related IR may help to explain the known association between obesity and malignancy and promotes IR and the harmful effects of compensatory hyperinsulinaemia as central to the pathogenesis of such tumours [15].

Given the close link between obesity and the development of PI3-K dysfunctional IR, it is important to explore how obesity mediates its effects on the insulin pathway. It seems likely that multiple and complex pathways are implicated, with inter-linking and multidirectional effects. It is beyond the scope of this concise review to provide a detailed exposition of the complexities of molecular mechanisms. Rather, we provide an overview of the current main theories that link obesity with IR.

**Chronic inflammation:** The close association between body weight and IR [12,16] is likely mediated, at least in part, through inflammatory pathways [17]. Weight gain and obesity causes changes in the release of key adipokines and cytokines from adipose tissue that in turn manifest in both paracrine and endocrine effects, with the latter from release into the plasma. These changes include increased release of leptin and plasminogen activator inhibitor-1 and reduced release of adiponectin [18]. These key changes result in a generalised low-grade inflammatory response, mediated through infiltration of macrophages and other immune cells into metabolic organs, such as white adipose tissue (WAT), skeletal muscle, liver and pancreas [19]. This process associates with a shift from a predominantly anti-inflammatory to a proinflammatory profile [20]. Macrophage release of proinflammatory cytokines (including interleukin-1β that activates the “NOD-, LRR- and pyrin domain-containing protein 3” (NLRP3) inflammasome) result in autocrine and paracrine effects that enhance IR [20]. This chronic low-grade inflammatory response also facilitates tumour cell motility and invasion [18].

**Mitochondrial dysfunction:** Obesity can overwhelm the capacity of eutopic WAT depots. In this scenario, lipid deposition occurs ectopically in nonadipose tissues, such as skeletal muscle and liver. In recent years, a great deal of evidence supports an important role of mitochondrial dysfunction in the development of obesity-related IR, stimulated through ectopic fat deposition [21]. Lipid-induced production of reactive oxygen species (ROS) within skeletal muscle promotes mitochondrial dysfunction and the development of IR [21]. Furthermore, a harmful feedback loop can occur whereby ROS production from impaired mitochondria results in further mitochondrial dysfunction and the worsening of IR [21]. Related to the mitochondrial dysfunction and production of ROS, obesity-induced endoplasmic reticulum (ER) stress and inflammation within the liver may also result in hepatic IR and gluconeogenesis [22].

***Gut microbiota dysbiosis:*** In recent years, there has been a transformation in our understanding of the gut microbiota and its role in health and chronic disease. A number of our data originate from rodent models. In a recent meta-analysis of data on changes in the gut microbiota in high-fat diet (HFD)-induced obese rodents, there was a demonstration of the structural and functional dysbiosis of the gut microbiota in HFD-induced obesity [23]. Compared with their lean counterparts, HFD-induced obese rodents increased Lactococcus and reduced Turicibacter, each consistent with an enhanced inflammatory response [23]. There was also an abundance of Dorea, Oscillospira and Ruminococcus. Furthermore, functional differences in the gut microbiota between lean and HFD-induced obese rodents occurred, with the latter manifesting metabolic pathways that converge on the biosynthesis of lipid and carbohydrate and short chain fatty acid (SCFA) metabolism [23]. From these data, it is not possible to implicate clearly gut microbiota dysbiosis as a mediator between obesity and IR. The gut microbiota has multiple and complex effects on appetite and metabolism and may influence body weight. Furthermore, we should be cautious about extrapolating data from rodent studies into humans. However, the rodent data outlined here provide compelling evidence that HFD-induced obesity associates with both structural and functional gut microbiota dysbiosis, consistent with the promotion of a proinflammatory state and the subsequent development of IR.

**Adipose extracellular matrix (ECM) remodelling:** In response to nutritional cues, adipocytes and their precursors need to change their shape, size and function. This process requires remodelling within a WAT ECM [24]. Weight gain, with its associated expansion of WAT depots, results in a localised hypoxic response: the expanded WAT essentially outstrips its own vascular supply and becomes hypoxic, resulting in the acceleration of fibrosis and inflammation within the expanding WAT [24]. In this scenario, the excessive deposition of ECM within WAT limits the angiogenic response. Given the regulation of insulin sensitivity by ECM receptors, such as CD44 and integrins, the accumulation of ECM within the WAT results in the worsening of IR [24].

In addition to the mechanisms outlined above, numerous other factors influence insulin sensitivity, some of which may be influenced in some way indirectly by weight gain (excess adiposity) and obesity. Amongst these factors include genetic abnormalities that affect the insulin receptor or the proteins of the cellular insulin pathway and fetal malnutrition [11]. Vitamin D may also influence IR and has received a great deal of attention in recent years. In one study on more than 2200 Greek schoolchildren aged between 9 and 13 years, the prevalence of the vitamin D insufficiency was significantly greater in children with obesity compared with in their overweight and normal weight counterparts (60.5% vs. 51.6%) [25]. Furthermore, children with IR had a 1.48-fold greater likelihood of vitamin D insufficiency compared with those without IR [25]. This study, as with much of the literature on the influence of vitamin D on IR, reported on the association data in which the proof of causality is lacking. Future studies should explore potential causal mechanisms linking vitamin D insufficiency with IR, and the degree to which obesity mediates the development of IR through effects on vitamin D. Possible indirect mechanisms include reduced sunlight exposure of the skin or increased sequestration of vitamin D within WAT with consequent reduced levels in the plasma.

To conclude this section, obesity-related IR appears confined to the PI3-K postreceptor insulin pathway that regulates the cellular metabolic effects of insulin. Obesity contributes towards the development of IR through numerous and complex mechanisms, which implicate changes in adipokines, cytokines, inflammatory response, mitochondrial dysfunction, ROS production, ER stress, gut microbial dysbiosis and remodelling of adipose ECMs. From this perspective, IR is located at a crossroad or gateway that functions as a key expedient linking weight gain and obesity with its metabolic and cognitive sequelae.

## 3. Role of IR in the Development of Metabolic Dysfunction

IR manifests with a reduction in the uptake and oxidation of glucose and reductions in glycogen synthesis and the ability to suppress lipid oxidation [26]. Through the impairment of the PI3-K insulin pathway, IR plays a key role in the development of metabolic dysfunction and underlies a cluster of cardiovascular and metabolic abnormalities referred to as the metabolic syndrome (including hypertension, dyslipidaemia and dysglycaemia). Furthermore, IR promotes weight gain related to secondary compensatory hyperinsulinaemia [27], with a resulting vicious cycle of worsening IR and its metabolic sequelae. Having explored the complex pathways that mediate effects of obesity on the development of IR, in this section, we provide an overview of the mechanisms by which IR contributes towards metabolic dysfunction.

**Hypertension:** IR results in compensatory hyperinsulinaemia that in turn may increase blood pressure via multiple mechanisms. These include enhanced renal sodium reabsorption, the hypertrophy of resistance blood vessels and the activation of the sympathetic nervous system [28,29]. The development of hypertension can also result in the worsening of IR through changes in the structure of the vasculature and the impairment of vasodilation within skeletal muscle, with the reduced delivery of glucose and insulin to skeletal muscle cells [28]. Interestingly, however, studies of animal models of hypertension suggest that IR actually precedes the onset of hypertension, consistent with a causal role of IR in the development of hypertension [30]. Furthermore, the data from healthy human volunteers suggest that changes in autonomic regulation influence insulin sensitivity. Autonomic imbalance may, therefore, result in both IR and hypertension, through effects on vasoconstriction [30]. Therefore, although there are close links between IR and hypertension, pathogenic mechanisms appear complex and incompletely understood. Further studies are required to provide clarity regarding the exact underlying pathophysiologic mechanisms.

**Dyslipidaemia:** IR results in enhanced hepatic flux of fatty acids originating from the diet and resistance to the antilipolytic effects of insulin within adipose tissue [31]. These alterations of lipid metabolism manifest with dyslipidaemia and a characteristic proatherogenic lipid triad consisting of high plasma levels of triglycerides, small and dense low-density lipoproteins and low plasma levels of high-density lipoproteins [32]. Furthermore, such IR-induced aberrations of lipid profile may contribute towards inflammatory responses within the endothelium.

**Dysglycaemia:** IR associates with imbalanced glucose metabolism and chronic hyperglycaemia that in turn can trigger oxidative stress and an inflammatory response [33]. The reduced peripheral uptake of glucose into skeletal muscle cells represents a cardinal feature of IR. In this context, compensatory hyperinsulinaemia due to the enhanced insulin release from pancreatic β-cells helps to mitigate the development of hyperglycaemia. Therefore, the development of T2D requires additionally a degree of pancreatic β-cell dysfunction [34]. Interestingly, the data from both physiological and epidemiological studies suggest that disordered insulin secretion from pancreatic β-cells may actually contribute towards the development of IR and subsequent T2D [34].

The metabolic consequences of IR outlined in this section often co-exist and together contribute towards endothelial dysfunction and, ultimately, the formation of atherosclerotic plaque. These pathogenic processes can eventually manifest with macrovascular events. In the case of the myocardium, IR also causes damage through multiple pathways that include altered delivery of substrates including free fatty acids [35], impaired regulation of substrate metabolism [36] and alteration of signal transduction [37]. Furthermore, IR as a primary pathogenic factor also underlies nonalcoholic fatty liver disease (NAFLD) [11]. Finally, IR likely plays a key role in complex pathogenesis that underlies PCOS through metabolic dysfunction that stems from selective IR within the PI3-K postreceptor insulin pathway [38] and deleterious effects of compensatory hyperinsulinaemia on ovarian function (accounting for both hyperandrogenic and reproductive manifestations from co-gonadotrophin steroidogenic [39] and preantral follicular arrest [40], respectively) [41,42,43].

## 4. Role of IR in the Development of Cognitive Dysfunction

Numerous factors influence cognitive function, including those of both genetic and environmental origin. As outlined earlier, obesity-associated IR associates with a generalised chronic low-grade inflammatory response. Unfortunately, the brain is not immune from such inflammatory effects that may ultimately increase the progression of cognitive decline and the risk of development of Alzheimer’s disease (AD) [44]. In normal physiology, the mediation of immune functioning within the brain occurs through microglial cells that facilitate neuroprotection. However, in the context of central inflammation associated with obesity and IR, these same microglial cells can transform into reactive microglia and become injurious to local neuronal tissue (including apoptotic effects), through the release of cytotoxic cytokines [45]. Other factors that influence cognitive function include the expression of polymorphic proteins within the brain, including most notably the expression of ApoE4, as a risk factor for the development of dementia and cognitive impairment in the elderly [46,47]. However, the data reported in the last few decades also promote the central insulin pathway as an important determinant of cognitive function.

Insulin was first identified in the brain in 1978 [48]. Since then, a plethora of studies have transformed our knowledge and understanding of centrally acting insulin. As in the periphery, central insulin acts through the insulin receptor (IRec), but with predominant effects on insulin-sensitive glucose transporters-1 and -3 (GLUT1 and GLUT3, respectively), rather than GLUT4 which is the glucose transporter found primarily in adipose tissue and skeletal muscle [49,50]. The distribution of IRecs (and co-localized insulin receptor substrate (IRS)) [51] within the brain provides insight into insulin function centrally [52]. Although IRec mRNA is located in neuronal cell bodies, the distribution of IRec receptor protein is heterogeneous and includes pyramidal cell axons, neuronal terminals and synapses [46,53,54,55]. Accordingly, the central expression of IRecs covers a wide distribution including neuronal and glial cell expression, especially within areas important for cognition such as the cerebral cortex, hippocampus, hypothalamus and olfactory bulb [46,56]. Interestingly, the density of central IRecs differs between embryonic and adult brains [57].

Insulin within the brain, originating from the pancreatic β-cells and reliant upon efficient IRec-mediated transport of insulin across the blood brain barrier (BBB) [58], plays an important role in cognition, including the facilitation of learning and memory in older people [59]. Underlying mechanisms that mediate the procognitive effects of insulin include the promotion of nerve cell growth and development as well as the regulation of neurotransmitter release [59,60]. Furthermore, insulin provides nutritional support to nerve cells [61] and regulates the expression of various enzymes implicated in the control of glucose metabolism, including choline acetyltransferase [53]. Insulin may also support higher brain functions such as learning and memory, through effects on hippocampal synaptic plasticity [62]. The support for this hypothesis stems from a rat model of T1D (induced by streptozotocin), in which both hippocampal synaptic plasticity and spatial memory ability are significantly impaired and corrected by the administration of insulin [63]. Although the mechanisms that underlie the links between central insulin signalling and synaptic plasticity remain incompletely understood, this may implicate the modulation of glutamatergic neurotransmission [64] and the stimulation of translocation of γ-aminobutyric acid (GABA) receptors to the plasma membrane, thereby increasing functional GABA receptor expression within the postsynaptic membrane [46,65].

Following this outline of the important role of central insulin in the support and facilitation of cognitive function, it is important to consider the effects of IR on cognition. Any impairment of the central insulin-signalling pathway appears to promote the advancement of cognitive dysfunction [66]. Indeed, the existing evidence suggests that IR is an independent risk factor for cognitive impairment and neurodegeneration [67]. This is consistent with a known epidemiological and biological link between T2D and AD [67]. The incidence of AD in patients with longstanding diabetes mellitus (DM) is twice that of normal elderly people [68]. Furthermore, disturbed brain insulin signalling mechanisms contribute towards the development of biochemical, molecular and histopathological lesions in patients with AD [46,67,69].

The underlying mechanisms that mediate cognitive decline in the context of IR are probably multifactorial. Central insulin signalling appears to depend on both the sensitivity of central IRecs and adequate levels of insulin within the brain. In the early stages of IR, secondary hyperinsulinaemia can result in the neuronal degeneration and irreversible impairment of memory through the chronic exposure of neurons to elevated levels of insulin within the brain [70]. However, over time, secondary hyperinsulinaemia can result in damage to the function of the BBB, with the subsequently reduced transport of insulin from the peripheral circulation into the brain parenchyma [71]. Indeed, in patients with AD, there are usually low levels of insulin within the brain and cerebrospinal fluid (CSF), with high levels of peripheral insulin [46,72]. Therefore, following initial neuronal impairment from the effects of central hyperinsulinaemia, there is a reduction in the levels of central insulin, despite peripheral hyperinsulinaemia. This scenario, compounded by central IR, often associates with increased levels of cerebral beta amyloid that further worsens central IR [73]. Finally, central IR also promotes tau protein phosphorylation [74] that contributes to the formation of neurofibrillary tangles that in turn contributes towards impaired cognition and ultimately AD pathology [46,75]. The combination of neuronal damage from early exposure to chronic elevations of central insulin, impaired BBB function and subsequent reduction in central insulin and central IR compounded by cerebral beta amyloid and tau protein phosphorylation creates a perfect storm for impaired central insulin action in the context of obesity-related IR. These mechanisms help to explain the association between T2D and AD [67] and the correlation between central obesity and impaired cognitive function in the elderly [76]. The data from animal studies implicate hippocampal IR as a mediator of cognitive dysfunction in patients with both T2D and AD [77]. To compound the effects of IR on obesity-associated T2D, cardiovascular risk factors (including hypertension and atherogenic dyslipidaemia) further contribute independently towards the development of cognitive impairment [78].

In support of an important role for insulin in cognitive function, various studies support the notion of insulin as a neuroprotector. In one study on normal rats, the intra-hippocampal injection of insulin significantly improved spatial memory ability [79]. Furthermore, the application of nasal insulin in a diabetic mouse model resulted in improved decline in cognitive function related to DM, in addition to changes in molecular pathology and brain morphology [46,80]. In another study, the use of the insulin sensitizer, pioglitazone, in a rat T2D model improved learning and memory function [81]. There are also data from human studies that suggest a beneficial effect of insulin administration on cognitive function, including improved memory function in patients with AD following insulin administration to maintain constant levels of glycaemia [82]. However, there are limitations of such human-based studies with peripheral administration of insulin, due to the confounding effects of the peripheral metabolic effects of insulin (including improved glycaemic control in DM). This important confounder limits any firm conclusions regarding the direct central effects of insulin on cognitive function in humans, with most of the supporting evidence derived from animal-based studies, as outlined above.

## 5. Reversibility of IR with Lifestyle Interventions

Although IR plays a central role in the complex pathogenesis of metabolic dysfunction and cognitive dysfunction in people who gain weight and develop obesity, the compelling data support an important effect of lifestyle factors on insulin sensitivity. Undoubtedly, some of the improvements in insulin sensitivity following the adoption of healthy lifestyle factors stem from the attendant weight loss and reduction of excess adiposity. However, unfortunately, the typical trajectory of weight change following lifestyle modification emerges with early weight loss, a plateau at around six months and subsequent progressive and insidious weight gain thereafter [9,10]. Nevertheless, despite the challenges associated with the long-term maintenance of body weight, the beneficial effects of improved lifestyle on insulin sensitivity occur even independently of changes in body weight. The current evidence promotes close attention to the optimisation of lifestyle in the effective management of obesity-related IR, as a means of improving future cardiovascular risk and overall wellbeing. In this section, we provide a concise overview of the effects of key lifestyle factors on the reversibility of IR and discuss the main implications for the clinical management of obesity.

**Physical activity:** Much evidence supports an important role for regular physical activity and exercise in the optimisation of insulin sensitivity and the converse association of sedentariness with IR [83]. Physical training potentiates the effects of physical exercise on insulin sensitivity through the optimisation of glucose transport and metabolism [83]. Therefore, the degree of physical activity versus sedentariness represents an important underlying determinant of the overall spectrum of insulin sensitivity within the population. In a meta-analysis that included 17 studies on the effects of physical exercise on IR in overweight and obese children and adolescents, compared with the control group, exercise (especially aerobic training) associated with significant reductions in fasting serum insulin levels and homeostasis model assessment of insulin resistance (HOMA-IR) [84]. Indeed, even nonstrenuous physical exercise appears to influence IR. In a randomised trial of women with T2D (*n* = 53) divided into exercise and control groups, it was shown that an eight-week exercise program that consisted of stretching and walking activities three times per week resulted in significant improvements in IR, including significant reductions in plasma glucose and insulin levels [85]. Furthermore, in addition to the evidence for the relatively short-term and immediate effects of physical activity on insulin sensitivity, other data supports longer-term effects. In one large retrospective study on more than 6800 Japanese adults aged between 40 and 79 years, it was shown that those who engaged in regular physical exercise throughout their teenage years into their 30s and 40s reduced their risk of IR (odds ratios (ORs) of 0.75 and 0.76 for men and women, respectively, following adjustments for other variables) [86]. Furthermore, there was a linear trend between the degree of regular physical exercise during younger years and the level of IR in middle and older age [86].

Although much evidence supports an important role of the physical activity and avoidance of sedentariness in the reduction of IR in both the short and longer terms, the underlying mechanisms that mediate such effects remain incompletely understood. In one study on skeletal muscle with comparisons between inherited IR and matched healthy controls and between trained and sedentary subjects, there were 12 genes identified that were associated with IR, in which the transcriptome profile reversed with exercise [87]. Furthermore, two IR-related genes (*MSTN* and *IL32*) were identified within muscle cells from both young and old subjects, providing insight into possible mechanisms by which exercise mediates metabolic benefits in humans with inherited IR [87]. However, more research is required to provide further insight into the underlying mechanisms that mediate effects of physical activity on insulin sensitivity.

**Diet:** Regarding the effects of dietary macronutrients on insulin sensitivity, some of our most compelling evidence relates to dietary fibre intake [88,89,90,91,92,93]. Our own group published the data from the highly phenotyped ProFiMet interventional study on 111 overweight adults with features of the metabolic syndrome, who were assigned randomly to one of four isoenergetic diets, each with a duration of 18 weeks [94]. These diets included high cereal fibre (HCF), high protein (HP), mixed high cereal fibre and protein (Mix). For the 84 participants who completed the dietary intervention, insulin sensitivity was significantly (25%) greater in the HCF group compared with those in the HP diet group [94]. We hypothesized that the attenuated effects of HCF on insulin sensitivity at 18 weeks probably resulted from the reduced dietary adherence towards the end of the trial [94].

Noninterventional observational studies corroborate the benefits of dietary fibre on insulin sensitivity. In one study from Mexico on more than 200 adolescents, following adjustments for age, sex, intake of saturated fatty acids and body fat percentage, those with the greatest dietary intake of fibre had a lower OR of 0.34 (95% CI (confidence interval): 0.13–0.93) and had an IR (defined by a HOMA-IR) of >2.97 [95]. Furthermore, a systematic review and meta-analysis on the application of dietary fibre and whole grains as a management strategy for patients with DM, a high-fibre diet associated with improved insulin sensitivity, and other aspects of metabolic health (lipid profile, HbA1C, body weight and C-reactive protein) [96]. However, despite these compelling data that support the beneficial effects of dietary fibre intake on insulin sensitivity, there were relatively few controlled studies (with a notable exception [93] that reported on the longer-term (>12 months) metabolic effects of dietary fibre intake [96]. This should be a focus for future research, to provide further evidence to promote the widespread adoption of a high-fibre diet within the populace [97].

The mechanism(s) by which dietary fibre mediates its metabolic benefits remains incompletely understood and contentious. Within the existing literature, current dogma implicates effects of dietary fibre on SCFAs. More relevant to soluble, fermentable fibre, at least some of the metabolic benefits probably do occur via effects on the gut microbiome, with subsequent release of SCFAs [97]. Through effects both locally within the ileum and following absorption across the ileal wall into the bloodstream, SCFAs may in turn influence the release of various gut hormones [98,99,100,101,102,103], adipokines [104], bile acids [105] and amino acid metabolic signatures [106], each of which may contribute towards overall insulin sensitivity. SCFAs may also reduce the leakiness of the gut wall itself, thereby limiting the absorption of endotoxins and the subsequent inducement of a generalised chronic low-grade inflammatory response that often associates with worsened IR [97].

However, the mechanisms by which dietary fibre exerts its metabolic benefits are likely to be more complicated than simply through effects on SCFAs. Dietary fibre generally consists of a mixture of both soluble and insoluble components. Interestingly, the predominant metabolic benefits (including the optimisation of insulin sensitivity and the associated reduced risk of the T2D development) appear to derive from the consumption of insoluble cereal dietary fibres and whole grains [97,107,108,109,110,111,112]. Conversely, soluble fibres appear to improve both overall lipid profile and the glycaemic index of carbohydrate-rich foods [91,113,114]. As outlined, although the mediation of some beneficial metabolic effects of soluble dietary fibre may occur through changes in SCFAs, interestingly, a high dietary intake of soluble, fermentable fibres does not associate with a reduced risk of developing T2D, in contrast to the increased dietary intake of insoluble, cereal fibres and whole grains [110]. The long-term studies in rodents have demonstrated similar data [115,116]. Therefore, an important role of SCFAs in conveying improved insulin sensitivity following the intake of insoluble (at best only moderately fermentable) fibres remains controversial [117]. Indeed, the relative lack of the fermentability of insoluble dietary fibre, despite its clear beneficial effects on improving insulin sensitivity and risk for the development of T2D, supports an underlying mediating mechanism alternate to that of SCFAs. Our own group proposed an inhibitory effect of dietary insoluble fibre on the absorption of dietary protein [94], subsequently corroborated by further reported data from numerous studies in humans and rodents, including interventional and prospective cohort studies [110]. Therefore, rather than changes in SCFAs, a more convincing explanation for at least some of the metabolically beneficial and protective effects (including improved insulin sensitivity) of dietary insoluble fibre (such as cereals and whole grains) is through the impairment of protein absorption.

Regarding the nonfibre effects of macronutrients on insulin sensitivity, there remains some controversy in the current literature. In a recently reported review on the effects of dietary modification on weight loss, no evidence supported any single dietary plan (including food group, dietary pattern or nutrient) as being more efficacious for weight loss than other options [118]. However, low-carbohydrate or low-glycaemic-index diets are associated with greater improvements in both IR and lipid profile [119]. Therefore, to optimise fully the dietary effects on insulin sensitivity, perhaps our best option is to ensure the combination of a high-fibre and low-carbohydrate diet.

**Sleep:** Sleep forms an essential component of human physiology. Unfortunately, sleep deprivation has become endemic in our modern society [120]. Sleep deprivation has far-reaching implications that extend well beyond IR and metabolic dysfunction [121]. In recent years, an abundance of data have emerged that provide compelling evidence to link sleep deprivation with increased risk of IR, obesity and T2D [120,122,123]. Interestingly, limited reported data implicate sleep fragmentation in the development of IR [124]. In a large study on more than 1000 European participants, the data derived from the relationship between insulin sensitivity and cardiovascular disease (EGIR-RISC) included self-reported sleep duration. There was a U-shaped association between sleep duration and IR, confirming an association of IR with extremes of sleep duration [125]. Furthermore, body mass index (BMI) was an important mediator between sleep duration and IR [125].

Although incompletely understood, the factors that mediate IR in response to sleep deprivation likely implicate central autonomic pathways, endocrine responses (e.g., changes in the key appetite hormones ghrelin and leptin) and inflammatory status [120,122]. However, the data from rodent-based studies suggest that changes in the gut microbiota in response to sleep deprivation may also mediate important effects on IR. In a study on mice that were exposed to sleep fragmentation for four weeks, there was increased food intake and reversible changes in the gut microbiota (including preferential growths of Lachnospiraceae and Ruminococcaceae and a reduction of Lactobacillaceae) [126]. Furthermore, sleep-fragmented mice had WAT inflammation and worsened IR, hypothesized to result from enhanced disruption to the colonic epithelial barrier [126]. Future studies should explore the effects of sleep duration on the human gut microbiota, as a potential novel therapeutic target for improved insulin sensitivity.

## 6. Conclusions

**Evidence-based conclusions:** Obesity represents a global threat to health. A large amount of the ramifications of obesity stem from its numerous indirect effects, including adverse effects on socio-economic aspects (e.g., taking sick leave from work, unemployment and reduced productivity and earnings), stigma (e.g., discrimination and social exclusion) and overall mental health, wellbeing and quality of life. Furthermore, the direct effects of obesity are substantial and include association with more than 50 obesity-related co-morbidities [127]. There are variable underlying pathogeneses for these numerous co-morbidities. These range from mechanical effects of obesity from pressure on weight-bearing joints, restrictions of breathing and restrictions of the upper airways in obstructive sleep apnoea to the effects of obesity-related societal views, prejudices and stigma on the development of depression and other mental health problems, including adverse impact on overall emotional wellbeing, self-esteem and general productivity [1]. However, despite these variable pathogeneses, IR represents a common theme that interweaves many of direct obesity-related co-morbidities, particularly relating to metabolic and cognitive effects [128]. In this way, IR sits at crossroads between weight gain (excess adiposity) and obesity and the metabolic and cognitive dysfunction that characterises and underlies many of obesity-related co-morbidities (Table 1).

Given the central role of IR as a key mediator in the deleterious effects of obesity, the successful modification of IR forms an important therapeutic strategy. Although weight loss associates with improved insulin sensitivity in the context of obesity, a key point is that the effective and potentially chronic improvement of IR can occur simply through the adoption of a healthy lifestyle. Of particular relevance to IR includes three pillars of lifestyle: (i) regular engagement in physical activity and avoidance of sedentariness; (ii) adoption of a healthy diet including high-fibre dietary intake; and (iii) improved sleep quality and sufficiency with the avoidance of sleep deprivation and disruption. Unfortunately, many of us in our modern-day world fail to achieve at least one of these healthy lifestyle components. Although there is popular engagement in physical activities in our leisure time, modern-day technology limits the need for many of us to engage in physically demanding tasks, at least in our work. Our modern-day highly processed and fibre-impoverished diets often stymie attempts at dietary modification [129]. Our modern-day “24/7” instant-access culture with busy and stressful lifestyles, noisy and light environments can have a negative impact on our sleep pattern. Furthermore, our culture and society does not seem to value sleep in the way that it should [130]. Indeed, as a pillar of a healthy lifestyle, sleep somehow often seems neglected from its other two counterparts, diet and exercise.

**Public health recommendations:** Although effective weight loss remains a key management strategy for obesity, the co-morbidities of obesity (mediated by IR) are also modifiable through effective lifestyle modifications. This would require a multifaceted approach in multiple settings (including schools) and address societal misconceptions regarding the benefits of physical activity and the avoidance of sedentariness [131], optimised dietary fibre intake [132] and the key principles of sleep hygiene and the importance of regular sleep sufficiency [133]. Key stakeholders can also make a difference, including food producers/companies and supermarkets/food retailers to optimise fibre contents of highly processed foods [97] and town planners and local councils to ensure the facilitation of pedestrian and cyclist transport [134]. Furthermore, cultural changes are required regarding the adoption of healthy lifestyles [135], particularly the value and importance of regular sleep sufficiency [133]. The effective population-wide implementation of these lifestyle modifications would benefit substantially the health of the nation.

With the grim era of the Covid-19 pandemic considered as another paradigm at the time of writing this review, it is easy to adopt a narrow-minded and struthious public health perspective and focus on one virus/disease at the expense of all other long-term health threats that affect our species. This is in no way to diminish the importance of the current Covid-19 pandemic and the herculean efforts to control the spread of this new virus, manage its sequelae and develop effective vaccines [136]. However, even in this context, aside from the older age, obesity (and associated IR) represents a major risk factor for both the susceptibility to and severity of the Covid-19 infection [137]. Therefore, the implementation of the lifestyle factors outlined here is particularly apt and important in the current era with the emergence of Covid-19 catalysing the urgency, with which we must act at a national level to improve our future health. It is also worth remembering this mantra for life: “when it is nobody’s responsibility, it is everybody’s responsibility”.

## Figures and Tables

**Table 1:** IR at the crossroad of obesity with associated metabolic abnormalities and cognitive dysfunction: factors that mediate effects of obesity on IR, the metabolic and cognitive sequelae of IR and the lifestyle reversibility of IR.

Factors that Mediate the Effects of Obesity on IR	Metabolic Sequelae of IR	Cognitive Sequelae of IR	Lifestyle Reversibility of IR
Chronic inflammation	Hypertension	Cognitive dysfunction	Physical activity and avoidance of sedentariness
Mitochondrial dysfunction	Dyslipidaemia	Learning deficiency	Optimised dietary fibre intake
Gut microbiota dysbiosis	Dysglycaemia	Memory impairment	Limited dietary carbohydrate intake
Adipose ECM remodelling	Nonalcoholic fatty liver disease & polycystic ovary syndrome	Impaired hippocampal synaptic plasticity	Optimised sleep sufficiency and quality

## Data Availability

Not applicable.
